# Impact of standardized endoscopic imaging on early detection rate of upper gastrointestinal tract cancer: A quasi-experimental study

**DOI:** 10.1097/MD.0000000000043927

**Published:** 2026-01-23

**Authors:** Zi-Xu Zhang, Wei-Yi Wang, Yi-Min Chu, Jin-Nian Cheng, Feng-Li Zhou

**Affiliations:** a Department of Endoscopy, Tongren Hospital, Shanghai Jiao Tong University School of Medicine, Shanghai, China.

**Keywords:** cancer, gastrointestinal, multicenter data, quasi-experimental, standardized endoscopic imaging

## Abstract

The outcomes of patients with upper gastrointestinal tract cancer are poor, and early detection is critical. This study investigated the impact of standardized endoscopic imaging on the early detection rate of upper gastrointestinal tract cancers. A quasi-experimental study was conducted between July 2022 and July 2023. Four tertiary hospitals were randomized to the control and standardized endoscopic groups. The physicians in the standardized endoscopic group underwent standardized endoscopy training and applied standardized endoscopic imaging procedures. The physicians in the control group applied routine endoscopic procedures. The numbers of early and advanced upper gastrointestinal tract cancers in both groups were determined and compared. In the control group, there were 1175 cases of upper gastrointestinal malignant tumors, including 39 cases of early esophageal cancer and 104 cases of early gastric cancer. The early endoscopic detection rate was 12.17% in the control group. In the standardized procedure group, there were 1184 cases of upper gastrointestinal malignant tumors, including 71 cases of early esophageal cancer and 182 cases of early gastric cancer, with an early detection rate of 21.36%. The detection rates with the standardized procedure were also higher for early-stage esophageal (14.86% vs 7.93%, *P* = .019) and gastric (25.78% vs 15.23%, *P* = .032) cancers. Using standardized endoscopic procedures may improve the early detection rate of esophageal and gastric cancers.

## 1. Introduction

Esophageal and gastric cancers are a global public health concern, and approximately half of the cases occur in China.^[1–4]^ The highest risk areas for upper gastrointestinal (GI) tract cancers are found in 2 geographic belts: an area from the Caspian Sea to northern China and an area on the eastern coast of Africa, from Ethiopia to South Africa.^[5]^ Although the age-adjusted incidence rate shows a slight decrease, gastric cancer incidence and mortality are relatively high in China.

The main reason for the high mortality is that esophageal and gastric cancers are frequently diagnosed in advanced stages, and their 5-year survival rate is about 30% in China.^[6–8]^ Therefore, early cancer detection is crucial for timely diagnosis and treatment, improving patient survival, reducing mortality, and enhancing quality of life.^[9]^ Unfortunately, the early detection rate of digestive tract cancers in China is only around 10%,^[8,10]^ much lower than in neighboring countries such as Japan and South Korea.^[11,12]^

Endoscopic screening is effective in the early diagnosis of upper GI cancers, resulting in timely treatment and reduced mortality.^[13]^ Still, endoscopy is influenced by the operator’s experience and skills, but the endoscopic diagnosis and early detection rate can be improved using standardized procedures.^[14,15]^ Consensuses must be developed to standardize the endoscopic procedures for diagnosing early upper GI cancers, and advocating learning and training on these standardized procedures will enhance the quality of endoscopic diagnosis and improve the detection of early upper GI cancers.^[16–19]^ Indeed, a recent study showed that the detection rates of adenoma and early cancer are still facing challenges,^[20]^ highlighting the need to promote standardized endoscopy methods in China.

Therefore, this study aimed to investigate the impact of standardized endoscopic imaging on the early detection rate of upper GI tract cancers.

## 2. Methods

### 2.1. Study design and participants

A multicenter quasi-experiment was conducted to investigate the use of standardized endoscopic procedures for the early detection of upper GI cancers. The study included patients diagnosed with upper digestive tract malignant tumors in 4 tertiary hospitals in China between July 2022 and July 2023. This study was approved by the Tong Ren Hospital, Shanghai Jiao Tong University School of Medicine (2022-039).

The inclusion criteria were patients with complete data and patients with histopathological confirmation of upper GI tract tumors. The exclusion criteria were: patients with postoperative recurrence, patients with confirmed non-early cancer cases by endoscopic ultrasound or postoperative pathology, or patients with tumor metastasis.

### 2.2. Intervention

The 4 tertiary hospitals included in the trial were randomized into 2 groups (the standardized procedure group and the control group) using a random number table. Senior endoscopists in the standardized procedure group received standardized endoscopic diagnostic and treatment procedure training and conducted endoscopic examinations accordingly. A standardized endoscopic upper GI tract examination procedure required a minimum of 18 images. A total of 4 images, including the greater curvature, lesser curvature, anterior wall, and posterior wall in the gastric antrum area, were necessary. Images of the gastric angle had to focus on the lesser curvature of the gastric angle and the positions of the anterior and posterior walls. Four images of the gastric fundus and cardia were required on the frontal view, anterior wall, posterior wall, and lesser curvature. Images of the lesser curvature of the gastric body, anterior wall, and posterior wall were needed. Four images were of the lesser curvature of the middle part of the anterior wall, the lesser curvature of the middle part of the posterior wall, the lesser curvature of the lower part of the anterior wall, and the lesser curvature of the lower part of the posterior wall. Images of the lower, middle, and upper parts of the greater curvature, and the frontal view of the gastric fundus are required. The final image was a standard vertical view of the gastric fundus. If lesions are identified, 4 images were taken from the distant view of the lesion, medium-range view, and close-range view before and after staining. Where possible, close-up images had to be taken for detailed observation.

### 2.3. Outcomes and definition

The diagnostic criteria for early esophageal and gastric cancers followed the “Chinese Consensus on Early Gastric Cancer Screening and Endoscopic Diagnosis and Treatment”^[10,21]^ and the “Chinese Expert Consensus on Early Esophageal Cancer Screening and Endoscopic Diagnosis and Treatment”.^[22,23]^ Early esophageal and gastric cancers are referred to as pathological changes limited to the mucosal and submucosal layers without lymph node metastasis.

The standardized endoscopic procedure was not used in the control group. The early detection rates of esophageal and gastric cancers between the 2 groups were compared. The early detection rate was calculated as the number of early malignant cases/ the total number of malignant cases × 100%.

### 2.4. Statistical analysis

SPSS 26.0 (IBM Corp., Armonk) was used for statistical analysis. The chi-squared test was used to compare the differences in rates or proportions between the 2 groups. All statistical tests were two-tailed, with *P*-values < .05 considered statistically significant.

## 3. Results

A total of 2359 cases of malignant tumors were identified, including 396 cases at the early stages. Among them, 1274 were male patients, and 1085 were female patients, with ages ranging from 27 to 93 years and a mean age of 47.5 years.

In the control group, there were 1175 cases of upper GI malignant tumors, including 39 cases of early esophageal cancer and 104 cases of early gastric cancer. The early endoscopic detection rate was 12.17% in the control group. In the standardized procedure group, there were 1184 cases of upper GI malignant tumors, including 71 cases of early esophageal cancer and 182 cases of early gastric cancer, with an early detection rate of 21.36% (*P* < .001). The rates of detection with the standardized procedure were also higher for early-stage esophageal (14.86% vs 7.93%, *P* = .019) and gastric (25.78% vs 15.23%, *P* = .032) cancers (Table 1).

**Table 1:** Comparison of early-stage and advanced cancer detection between the control and standardized procedure groups.

	Standardized procedure group	Control group	*P*
Malignant tumor detection, n	1184	1175	
Early-stage cancer detection, n (%)	253 (21.36)	143 (12.17)	<.001
Esophageal cancer, n	478	492	
Early-stage cancer detection, n (%)	71 (14.86)	39 (7.93)	.019
Gastric cancer, n	706	683	
Early-stage cancer detection, n (%)	182 (25.78)	104 (15.23)	.032

## 4. Discussion

This quasi-experimental study investigated the impact of standardized endoscopic imaging on the early detection rate of upper GI tract cancers. The physicians in the standardized endoscopic group underwent standardized endoscopy training and applied standardized endoscopic imaging procedures. The physicians in the control group applied routine endoscopic procedures. The numbers of early and advanced upper GI tract cancers in both groups were determined and compared. The early endoscopic detection rate was 12.17% in the control group compared with 21.36% in the standardized procedure group (*P* < .001). Similar improvements were observed for early-stage esophageal and gastric cancers. These results suggest that using standardized endoscopic procedures significantly improves the early detection rate of esophageal and gastric cancers in 4 Chinese hospitals.

Esophagogastroduodenoscopy is the most reliable screening method for detecting cancer lesions in the esophagus, stomach, and duodenum, especially early-stage cancer lesions that are occult to imaging methods.^[24,25]^ Although advances in endoscopic technology and techniques facilitate capturing and storing images, the standardization of endoscopic procedures and the acquisition of images to increase the detection rate of lesions, particularly early-stage ones, are important for cancer screening.^[26–28]^ In the present study, the early detection rate was higher in the standardized procedure group, where the trained endoscopists used standardized endoscopic imaging procedures, than in the control group. The standardized procedure involves a complete examination of the esophagus and stomach, with abundant photographic documentation of all parts. These are 2 central recommendations of the 2024 American Gastroenterological Association clinical practice update.^[28]^ Indeed, Best Practice Advice #5 states that ensuring sufficient upper GI endoscopy inspection time is necessary for complete mucosal visualization.^[28]^ A longer inspection time involves a higher likelihood of a complete examination of all parts of the stomach. Previous studies support that longer and more complete inspections are associated with higher detection rates of significant pathologies.^[29–31]^ Best Practice Advice #6 states that “A high-quality upper endoscopic examination includes a standardized photodocumentation protocol of anatomic stations,” including additional photos of suspicious abnormalities ^[28]^. Previous studies also support that a more complete photographic documentation protocol is associated with better detection rates.^[27,32–34]^

A previous study optimized the endoscopic procedure for early detection of upper GI cancers using a quality-assured bowel cancer screening protocol.^[35]^ Training in standardized endoscopic procedures improved the cancer detection rate among certified endoscopists.^[36]^ Using standardized procedures in artificial intelligence-assisted endoscopy exhibited higher accuracies in detecting early upper GI cancers.^[15,37,38]^ Advances in endoscopic technology and techniques can improve the early detection rate of upper GI cancers. Similarly, the early detection rate was greater when the advanced standardized endoscopic procedure was used in the current study.

This study had some limitations. Patient characteristics such as age, sex, socioeconomic status, *Helicobacter pylori* infection, family history, smoking, drinking habits, diet, and obesity can influence the early detection rate of upper gastrointestinal tract cancers.^[39–43]^ Unfortunately, the present study was performed at the hospital level, and patient-level characteristics were not fully collected, which may compromise the internal validity of our findings. Hospital case volume can influence the early detection rate of upper gastrointestinal tract cancers,^[37,44]^ but all 4 included hospitals were tertiary hospitals and reference centers for upper gastrointestinal tract cancer screening. The study was conducted from 2019 to 2021. The endoscopic technology might have changed over the 3 years, and this difference may have influenced the outcome, although a standardized endoscopic procedure was adopted in the standardized procedure group. Still, a similar bias could have affected the control group. The study did not consider geographical parameters, such as areas of high incidence, areas of modest incidence, and regions of low incidence, and therefore, the extrapolation of the outcome is limited. Future studies should collect detailed patient-level data and consider regional incidence rates to address these limitations and yield more robust, widely applicable conclusions.

## 5. Conclusion

In conclusion, using standardized endoscopic procedures may improve the early detection rate of esophageal and gastric cancers. Developing a nationwide consensus on standardized upper GI endoscopic procedures would help in the early detection of cancers and facilitate timely interventions.

## Author contributions

**Conceptualization:** Zi-Xu Zhang, Wei-Yi Wang.

**Data curation:** Wei-Yi Wang.

**Formal analysis:** Wei-Yi Wang, Yi-Min Chu.

**Funding acquisition:** Zi-Xu Zhang.

**Investigation:** Yi-Min Chu.

**Resources:** Jin-Nian Cheng.

**Software:** Jin-Nian Cheng.

**Writing – original draft:** Zi-Xu Zhang.

**Writing – review & editing:** Zi-Xu Zhang, Feng-Li Zhou.
